# Estimation of the herd-level basic reproduction number for African swine fever in Vietnam, 2019

**DOI:** 10.14202/vetworld.2022.2850-2855

**Published:** 2022-12-15

**Authors:** Thi Ngan Mai, Thanh Trung Nguyen, Viet Anh Vu, Thi Ngoc Vu, Thi My Le Huynh

**Affiliations:** 1 Department of Veterinary Microbiology and Infectious Diseases, Faculty of Veterinary Medicine, Vietnam National University of Agriculture, Hanoi, Vietnam; 2 Department of Pharmacology, Toxicology, Internal Medicine and Diagnostics, Faculty of Veterinary Medicine, Vietnam National University of Agriculture, Hanoi, Vietnam; 3 Central Laboratory, Faculty of Animal Science, Vietnam National University of Agriculture, Hanoi, Vietnam

**Keywords:** African swine fever, basic reproduction number, indoor production, Vietnam

## Abstract

**Background and Aim:**

African swine fever (ASF) is a notifiable viral disease of pigs and wild boars that causes severe economic losses to the swine industry. The pig industry in Vietnam was recently attacked by the ASF virus (ASFV) for the first time in history. However, we lack information regarding the transmissibility of ASF within indoor production systems communities, such as those in Vietnam. Therefore, we aimed to estimate the basic reproduction number (R_0_) for ASF during the early stages of transmission between farms in indoor production system communities from local and national data in Vietnam.

**Materials and Methods:**

The linear regression model approach for the susceptible infectious method was used in this study to estimate the transmission rate and, consequently, the R_0_ value.

**Results:**

The R_0_ values between-farm of ASF ranged from 1.41 to 10.8 in different scenarios of infectious period duration, from 15 to 30 days at the national and local levels.

**Conclusion:**

These results help to understand the scale and speed of ASF infection in Vietnam and to provide a scientific basis to implement control measures to restrict the spread of ASFV in other locations.

## Introduction

African swine fever (ASF) is a highly lethal and contagious disease affecting pigs worldwide. The disease is caused by the ASF virus (ASFV), a large double-stranded DNA virus and the sole member of the family Asfarviridae [1]. African swine fever poses a severe threat as pigs are a primary source of animal protein for food supply and directly support the livelihoods of farmers and stakeholders in many countries. In the early 1900s, ASF was first described in Kenya as an acute hemorrhagic fever in domestic pigs with a case fatality rate approaching 100% [2]. African swine fever virus subsequently spread through domestic pig populations in most sub-Saharan countries, reaching West Africa in the 1950s [3]. In 1960, ASFV genotype I was detected in Portugal and Spain and then in several European countries. This genotype was eradicated 35 years later, and since then, only Sardinia has been affected by genotype I, which is now endemic on the island [4]. In 2007, the ASFV genotype II strain was introduced in Georgia and subsequently spread throughout Europe, reaching Italy a few months ago. African swine fever virus had also concurrently spread to Spain, following other European and American countries other than Georgia, mainly through swill feeding [5]. In August 2018, ASFV was detected in China, the leading pig producer worldwide [6]. Since then, the virus has also been reported in several Asian countries, such as Vietnam, Mongolia, Cambodia, North Korea, South Korea, the Philippines, and Timor-Leste [7]. In Vietnam, ASF was first recorded in early February 2019 on small farms in the Hung Yen and Thai Binh Provinces, and then spread rapidly in the northern and eventually in all 63 provinces of Vietnam [8]. Most cases of ASF during the early disease phase in Vietnam were observed in small-scale farms in the northern part of the country, where pig production, with its unique sociological and cultural practices, mainly occurs.

At present, disease control measures typically include culling large numbers of pigs and strict biosecurity measures [7]. The continuing spread of ASFV and ineffective control measures pose a serious threat to the global pig industry and food security. Each farming system has its distinct risk factors, including high levels of movement of people around conflict regions, the lack of good farming practices, and biosecurity [9]. In the domestic population, ASFV can be transmitted by direct or indirect contact with infected animals through short-distance aerosols, fomites, and human and pig movement networks; however, their role as vectors in the potential transmission pathway has not been elucidated [10]. In addition, small-scale farms implemented indoor production systems with less strict biosecurity measures compared with that in large-scale industrial farms. We currently lack information regarding the transmissibility of diseases such as ASF within indoor production systems communities, such as those in Vietnam.

The basic reproduction number (R_0_) is essentially used to express an average number of secondary infections caused by one infected individual in a fully susceptible population during its entire infectious period. The disease will disappear from the population if R_0_ is below 1; otherwise, the disease may still be able to spread in a population [11]. It is one of the elemental parameters used in underpinning rational control strategies based on disease modeling. It quantifies the spread of infectious disease and gives information on the ability of a pathogen to spread in a vulnerable population, from which the required vaccination coverage for disease control can be derived. Therefore, the R_0_ value provides a better understanding of the dynamics of infectious disease outbreaks and the development of effective disease control measures [12]. Some studies have demonstrated quantifying the basic reproductive number of ASFV between farms [13–15]. However, these studies have only been conducted in free farming systems, private backyards, subsidiary holdings, wild boars, or not during an epidemic.

Therefore, we aimed to quantify the ASFV transmission through an R_0_ estimate between farms in indoor production systems communities during the early epidemic stage from local and national data in the context of Vietnamese outbreaks.

## Materials and Methods

### Ethical approval

This type of study does not require ethical approval.

### Study period and location

This study was conducted during the early stages of ASF occurrence, from February to August 2019. All the data are from confirmed outbreaks that occurred in some northern provinces (Hung Yen, Thai Binh, Thai Nguyen, Quang Ninh, and Hai Duong) and the rest of Vietnam.

### Data source

**Figure-1:**
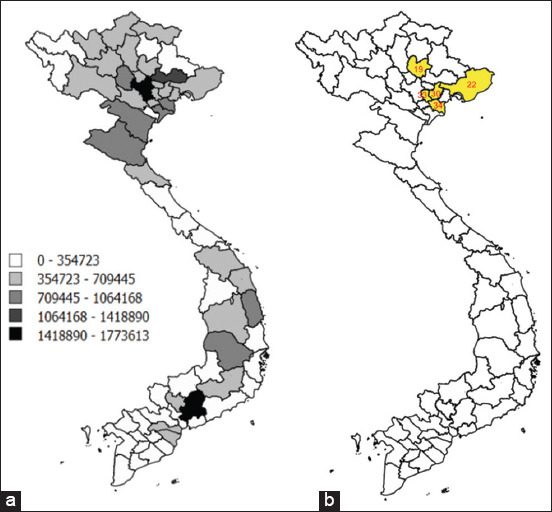
Spatial distribution of pigs in Vietnam and the geographical locations of Hung Yen, Thai Binh, Thai Nguyen, Quang Ninh, and Hai Duong Provinces in northern Vietnam. (a) Pig density is illustrated by deepening shades of black, indicating category level. (b) The geographical locations of Hung Yen, Thai Binh, Thai Nguyen, Quang Ninh, and Hai Duong Provinces are highlighted in yellow [Source: The geographical locations of farms were mapped using a free and open source Quantum Geographic Information System (QGIS) version 2.14.14 (https://www.qgis.org/en/site/)].

This study was based on data from confirmed outbreaks that occurred in some northern provinces (Hung Yen, Thai Binh, Thai Nguyen, Quang Ninh, and Hai Duong, highlighted in yellow color) and the rest of Vietnam (Figure-1) during the early stages of ASF occurrence, from February to August 2019. Local data of ASF outbreaks in Thai Binh, Hung Yen, Thai Nguyen, Quang Ninh, and Hai Duong Provinces were reported to their respective Animal Husbandry and Veterinary sub-departments under the Department of Animal Health, Vietnam. National data for this study were collected directly from the department of animal health. These data also formed part of the basis for international reporting to the World Organization for Animal Health.

A farm, defined as one or more buildings located in close geographical proximity under the same ownership with animals managed as a single population, was considered the epidemiological unit. Thus, our estimates of R_0_ reflected the spread between herds. All outbreaks during the study period were assumed to have been reported, and all herds were susceptible to the disease during the same period. The exact number of herds present during the study period could not be determined directly; therefore, it was estimated from the data on the distribution of livestock in Vietnam in 2019 from the General Statistics Office of Vietnam.

### Estimation of the transmission rate (β) at between-farm level

In this study, we estimate the daily transmission rate (β) for ASFV transmission between herds of pigs based on data from confirmed outbreaks, using the compartmental susceptible infectious removed (SIR) model. This was used to estimate β using a linear regression model (LRM) from the epidemic data. The regression model was defined as follows:

log (−log[1−C/S]) = log (β) + log (IΔt/N)

Where C, S, and I indicate the number of newly infected herds, susceptible herds, and infectious herds at the start of the time interval Δt, respectively. The infected farms (I) were assumed to be infectious until all pigs were dead or culled.

These estimates were performed using the bootstrapped technique with 1000 iterations, and their mean was considered an estimate of β with a 95% confidence interval (CI).

### Estimation of the basic reproductive number (R_0_) at the between-farm level

R_0_ was estimated from β and the infectious period of the herd (T). To reflect the uncertainty of this parameter, three values from the infectious period in a farm were obtained from the following published works: 15 days [13], 19 days [14], and 30 days [15]. The median value of R_0_ and the 95% CI limits were established for each of the three T scenarios.

### Statistical analysis

The standard Microsoft Office Excel 2010 package was used for data processing, running the regression model, and performing the bootstrapped technique. Quantum Geographic Information System version 2.14.14 (https://www.qgis.org/en/site/) was used for geovisualization.

## Results

### Data collection

**Figure-2:**
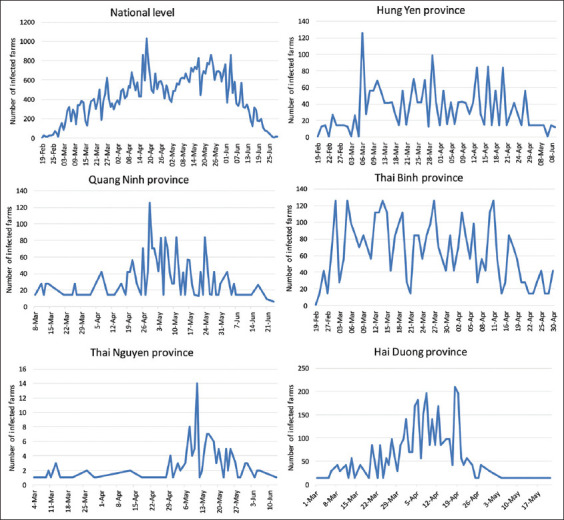
Farms infected daily with African swine fever in the national provinces of Hung Yen, Thai Binh, Hai Duong, Thai Nguyen, and Quang Ninh, respectively, from February to July 2019.

Following data collection, ASF resulted in 54,175 cases in farms in the whole country from February to July 2019. The distribution of daily infected farms is presented in Figure-2. In addition, 2,962,573 pig herds were estimated to be present at the national level, as well as 23,387, 108,623, 90,075, 40,643, and 35,387 herds present at the local level in Hung Yen, Thai Binh, Thai Nguyen, Quang Ninh, and Hai Duong Provinces, respectively, calculated from data obtained from General Statistics Office of Vietnam [16].

### Between-farm transmission rate (β)

Using the LRM approach from the SIR modeling method, the estimate for the daily transmission rate between farms at the national and local levels is calculated and recorded in Table-1. These values were 0.165 (95% CI: 0.159–0.172) at the national level and 0.30 (95% CI: 0.29–0.31), 0.36 (95% CI: 0.34–0.37), 0.105 (95% CI: 0.104–0.107), 0.094 (95% CI: 0.092–0.096), and 0.129 (95% CI: 0.128–0.131) at the local province level in Hung Yen, Thai Binh, Thai Nguyen, Quang Ninh, and Hai Duong, respectively. These data indicated that the between-farm transmission rate (β) values in Hung Yen and Thai Binh were higher than that at the national level. Moreover, the β value was highest in Thai Binh compared with the other provinces.

**Table-1:** Estimated values of transmission rates (β) at the between-farm level of ASFV.

Transmission rate (β)	Province code	95% CI		

		Value	Lower limit	Upper limit
National	-	0.165	0.159	0.172
Hung Yen Province	33	0.30	0.29	0.31
Thai Binh Province	34	0.36	0.34	0.37
Thai Nguyen Province	19	0.105	0.104	0.107
Quang Ninh Province	22	0.094	0.092	0.096
Hai Duong Province	30	0.129	0.128	0.131

ASFV=African swine fever virus, CI=Confidence interval

### Basic reproduction number (R_0_) at the between-farm level

Transmission rate values (R_0_) between farms at the national level are estimated and recorded in Table-2, along with the R_0_ values of the local provinces. These consist of transmission rate values resulting after an infectious period of 15, 19, and 30 days, respectively (Table-2). Notably, the R_0_ values were 2.48 (95% CI: 2.39–2.58) and 4.5 (95% CI: 4.35–4.65), 5.4 (95% CI: 5.1–5.55), 1.58 (95% CI: 1.56–1.61), 1.41 (95% CI: 1.38–1.44), and 1.94 (95% CI: 1.92–1.97) at national and Hung Yen, Thai Binh, Thai Nguyen, Quang Ninh, Hai Duong Provinces, respectively, as the infectious period is 15 days. For an infectious period of 19 days in affected farms, the between-farm R_0_ was estimated at 3.14 (95% CI: 3.02–3.27) and 5.7 (95% CI: 5.51–5.89), 6.84 (95% CI: 6.46–7.03), 2.00 (95% CI: 1.98–2.03), 1.79 (95% CI: 1.75–1.82), and 2.45 (95% CI: 2.43–2.49) at national and Hung Yen, Thai Binh, Thai Nguyen, Quang Ninh, Hai Duong Provinces, respectively. In addition, the between-farm R_0_ was 4.95 (95% CI: 4.27–5.16) and 9.0 (95% CI: 8.7–9.3), 10.8 (95% CI: 10.2–11.1), 3.15 (95% CI: 3.12–3.21), 2.82 (95% CI: 2.76–2.88), and 3.87 (95% CI: 3.84–3.93) at national, Hung Yen, Thai Binh, Thai Nguyen, Quang Ninh, Hai Duong Provinces, respectively, from the infectious period was 30 days. The highest R_0_ values were in the Thai Binh Province, and the lowest R_0_ values were estimated from the national data. These results suggested that the estimations of the basic reproduction number varied considerably across the different scenarios for the different durations of the infectious period.

**Table-2:** Estimated values of basic reproductive numbers at the national and local between-farm level of ASFV.

Location	Province code	Transmission rate (R_0_)		

		15 days	19 days	30 days
National	-	2.48	3.14	4.95
Hung Yen Province	33	4.50	5.70	9.00
Thai Binh Province	34	5.40	6.84	10.80
Thai Nguyen Province	19	1.58	2.00	3.15
Quang Ninh Province	22	1.41	1.79	2.82
Hai Duong Province	30	1.94	2.45	3.87

ASFV=African swine fever virus

## Discussion

Estimates of the basic reproduction number are fundamental in underpinning rational control strategies based on disease modeling. However, we lack studies on the estimate of the R_0_ from field data in the endemic regions of predominantly small-household indoor production in Vietnam. In this study, we estimated R_0_ between farms using national and local epidemic data collected between February and August 2019 from pig farms that were attacked by the ASFV during the early stages of the epidemic in Vietnam.

In this study, the SIR method was applied to estimate β using an LRM from ASF epidemic data. The LRM technique has been described and used in several studies for R_0_ estimations [13, 15, 17]. Therefore, we believed that the method would also provide our study with an accurate estimation of R_0_ because most of the cases were reported in the high-density area of pig farms in Northern Vietnam during the early phase. The between-farm R_0_ values of ASF were estimated from the respective transmission rate values to range from 1.41 to 10.8 in different scenarios of infectious period duration, from 15 to 30 days at national and local levels. Despite uncertainties surrounding the infectious period duration, empirical data from epidemics can be a valuable source for estimating epidemiological parameters. Some studies have been conducted to estimate the R_0_ of ASF between farms [13–15]. In these studies, data from 168 cases from 2007 to 2010 were used to estimate R_0_ values between 2 and 3 in wild boars and domestic pigs, 1.63–3.24 in free-range pig production systems, and 1.65 in private backyards or subsidiary holdings production systems.

Most of our estimations are higher than these reported values, which could be attributed to the different production systems present in the study and were captured during the early stages of ASF outbreaks. The R_0_ value might change due to different conditions such as populations, pig species, type of data available, swine population density, and calculation methods [13, 18–20]. In Vietnam, the pig production systems practiced in the local provinces are indoor production systems, predominantly small-scale farms with less strict biosecurity measures. Although medium- and small-scale farms are primarily operated by individual farmers, they are closely connected. Therefore, moving pigs from private farms to company farms is exceedingly rare. In the context of intensive connections between private farms, there tends to be a higher risk of cross-contamination for private farms than company farms through animals, vehicles, and human contact. Moreover, the lenient biosecurity measures applied in small farms compared with those in large-scale industrial farms are an additional factor in promoting virus transmission. A previous study has indicated that the spread of diseases can be reduced by applying strict biosecurity measures [21]. Therefore, the R_0_ values derived from the different studies and geographical areas are comparable to a limited extent. Due to the limited assessed regions, data from more locations should be examined in a future study to better understand the epidemiology of ASF in Vietnam. In addition, the length of the infectious period has a negligible effect on the relative ranking of basic reproductive number estimates nationally and for Hung Yen and Thai Binh Provinces. Therefore, details regarding the infectious period should be disregarded during decision-making. Concordantly, a recent study reported that the estimations of the daily transmission rate changed over time and different epidemic curves for severe acute respiratory syndrome revealed similar impacts of control measures [22].

Notably, the R_0_ values for the data at the national level are lower than those for the provincial-local areas such as Hung Yen, Thai Binh. In addition, the highest number of cases for the different levels was reached on different dates; March 2 and 6 in Hung Yen and Thai Binh localities, respectively, and April 18 nationwide (Figure-2). The aforementioned provinces are located in the northern part of the country, where most piglets are produced and transported to Southern Vietnam [23]. The main economic activity in these provinces is subsistence agriculture.

In Vietnam, ASF was first reported in Hung Yen and Thai Binh Provinces in February 2019, and then spread rapidly across the northern areas and the whole country [8]. As the first cases were detected in Vietnam, they also appeared to have an effective dispersion rather than a rapid one. In addition, as Hung Yen and Thai Binh were affected earlier, their control measures were likely applied less adequately compared with those in provinces with delayed incursions of the disease. Consequently, the R_0_ value in these two provinces is higher than the R_0_ value in the rest of the country. Moreover, the R_0_ values were higher in the Thai Binh Province than in the Hung Yen Province. This might be attributed to the higher livestock density in Thai Binh compared with that in the Hung Yen Province (Figure-1). Pig population density is one of the critical risk factors that may have also influenced R_0_ values [13]. The higher density of pig farms would increase the number of contacts between farms through the movements of pigs, persons, vehicles, or equipment [24].

Decision-making strategies to be implemented during an epidemic are complex, usually involving technical, political, sociocultural, and economic issues. At present, controlling an ASF outbreak by culling the infected herds and quarantining the affected areas is only feasible in developed countries with sufficient economic support to compensate farmers [25]. In resource-constrained countries such as Vietnam, the only feasible measures are to focus on preventive mitigation, including enhanced biosecurity and early detection and response. Estimates of R_0_ provide a means to better understand the dynamics of infectious disease outbreaks and to assess the efficacy of disease control measures. A previous study used the estimate of between-farm R_0_ to determine the level of herd depopulation required to curb the spread of ASF in at-risk regions [14].

In the SIR model, we assume homogeneous mixing of the population; all individuals in the population are assumed to have an equal probability of encountering one another. This does not reflect population structures, where most contact occurs within the limited networks. The limitation of the SIR model is that it does not account for the role of reducing connectivity and movement controls during the outbreak. Another limitation is that all outbreaks during the study period are assumed to have been reported. However, reporting delays or withholding of information could happen during the epidemic period due to the support policy from the government for culling infected herds. Nonetheless, our estimation was based on the confirmed cases, which provide a relatively accurate approximation that is valuable for understanding the epidemiology of ASF in Vietnam.

## Conclusion

This is the first study that estimates the R_0_ values using national and local epidemic data during the early stages of the epidemic of indoor pig production systems in the context of Vietnam ASF outbreaks. These results may contribute to a better understanding of the epidemiology of ASF in Vietnam and provide a scientific basis for implementing control measures to restrict the spread of ASFV in other localities.

## Authors’ Contributions

TNM, TTN, and TMLH: Conceived, designed, and supervised the study. VAV and TNV: Collected the data. TNM, TTN, and TMLH: Analyzed the data and edited the final manuscript. All authors have read and approved the final manuscript.
